# Consequences of the Reproductive Effort of Dioecious *Taxus baccata* L. Females in a Generative Bud Removal Experiment—Important Role of Nitrogen in Female Reproduction

**DOI:** 10.3390/ijms232214225

**Published:** 2022-11-17

**Authors:** Mariola Rabska, Marian J. Giertych, Kinga Nowak, Emilia Pers-Kamczyc, Grzegorz Iszkuło

**Affiliations:** 1 Institute of Dendrology, Polish Academy of Sciences, Parkowa 5, 62-035 Kórnik, Poland; 2 Institute of Biological Sciences, University of Zielona Góra, Prof. Z. Szafrana 1, 65-516 Zielona Góra, Poland

**Keywords:** nitrogen, carbon, resource allocation, reproductive effort, *Taxus baccata*, dioecious, bud-removal experiment

## Abstract

Dioecious species differ in the pattern and intensity of male and female reproductive investments. We aimed to determine whether female shoots deprived of generative buds show biochemical features, indicating their less-pronounced reproductive effort. For this purpose, the same branches of mature *Taxus baccata* females were deprived of generative organs. In the second and third years of the experiment, measurements were made in every season from the control and bud-removed shoots of females and control males. Bud removal caused an increase in nitrogen concentration almost to the level detected in the needles of male specimens, but only in current-year needles. Moreover, differences between male and control female shoots were present in the C:N ratio and increment biomass, but they disappeared when bud removal was applied to females. Additionally, between-sex differences were observed for content of phenolic compounds, carbon and starch, and SLA, independent of the female shoot reproductive effort. The study revealed that nitrogen uptake in seeds and arils may explain the lower nitrogen level and consequently the lower growth rate of females compared to males. At the same time, reproduction did not disturb carbon level in adjacent tissues, and two hypotheses explaining this phenomenon have been put forward.

## 1. Introduction

Females of dioecious woody species, in most cases, exhibit greater reproductive effort than males [1,2,3], as they produce not only flowers or strobili but also seeds and accompanying structures, such as arils. Females’ reproductive effort manifests itself, for example, in the reduction of the growth rate after maturation [4,5]. However, real differences between sexes in reproductive effort can sometimes be seen only in the whole plant or at the population level because of the cumulative effects of reproduction [4].

In addition to differential reproductive effort, the sexes of dioecious species also show differences in compound allocation, not only in their distribution but also in their temporal accumulation [6]. Differences in secondary compounds accumulation and transport in the sexes can also be a part of the response to various environmental conditions [7]. Moreover, the accumulation of different compounds in plants can change with the age of leaves (needles), with greater accumulation of phenolic compounds, and lower accumulation of nitrogen [8] and phosphorus [8,9] in older leaves.

Generative organs production has a great cost for the whole plant. Numerous studies have shown that plants with suppressed reproductive investment increase their vegetative biomass (e.g., [10,11,12,13]), but it can depend on plant genotype [14] and phenology [15]. However, in some cases, a reduction in dry matter accumulation as a response to suppressed reproduction can also be observed [16]. Flower bud removal experiments, despite some drawbacks (see [17,18]), are widely applied to assess plant investment in reproduction. Flower bud removal leads to a series of changes in plant tissues. The removal of reproductive structures can inter alia cause an increase in starch concentration [19], greater allocation to the rhizome [11,20], and reduction in RUBISCO activity [21]. Moreover, the removal of flower buds results in increased nitrogen uptake [22] and nitrogen and carbon storage [23]. It can also prevent N loss in the source leaves of flowers [24]. However, the duration of the experiment also matters, as Ehrlén and Van Groenendael [11] observed that long-term removal of generative organs resulted in increased vegetative size and a higher probability of flowering and setting fruit, but those effects were absent in short term experiments. The removal of flower buds can increase future flower yields [25]. Moreover, it is expected that control plants, in comparison to bud-removed plants, increase photosynthetic assimilation, as their demand for carbon is greater [26,27], but plants can also gain additional resources by increasing uptake or having more efficient use [28]. However, Nicotra [29] found that flower removal increased the photosynthetic capacity of female *Siparuna grandiflora* during flowering and fruiting. In some cases, compensation mechanisms, such as the response to flower bud removal, exist, but they depend on the plant’s features. Cross and Burgess [30] discovered that greater plants with greater fruit production were able to increase; for example, mean fruit weight in response to flower bud removal and compensation was not observed in plants with smaller sizes and smaller yields.

Nevertheless, many studies on dioecious species show that the production of generative buds is established at the expense of vegetative shoot growth in both sexes, and this effect can last longer than the current growing season [28]. However, the differential influence of generative organ removal on both sexes has also been observed. Elmqvist et al. [3] showed that, in willow, generative bud removal results, inter alia, in the production of greater shoots in females but not males. Conversely, Teitel et al. [31] found that reproductive allocation suppresses vegetative growth to a greater degree in males than in female *Rumex hastatulus*. Milla et al. [32] found that in females of *Pistacia lentiscus*, there was no trade-off between fruit load and current-year vegetative growth. Similar results were found by Turcotte and Houle [33] for *Salix planifolia*, with a lack of trade-off between growth and reproduction and Cipollini and Whigham [34] for *Lindera benzoin*, where partial fruit removal resulted in a trade-off in future fruit production but not in growth and element concentration. Similarly, in willow, there is an influence of suppressed reproduction on future reproduction investment in females [17,33,35]. Flower bud removal experiments can be used in dioecious species to assess different reproductive efforts [36] and compensation mechanisms [27].

Leaves placed near generative structures increase their photosynthesis to supply generative structures with carbon [37,38,39]. Plants can reduce delayed reproductive costs by biomass allocation, as well as compensation mechanisms, such as an increase in photosynthetic assimilation [40]. Plants compensate for their greater reproductive effort by increasing photosynthesis in comparison to those with their generative buds removed [26,27], and removal of leaves can lead to increased photosynthetic rates in remnant leaves (even overcompensating for leaf loss) (reviewed in [41]). An increase in photosynthesis as a compensation mechanism, in turn, can result in greater water and nitrogen demand [42]. Nitrogen is an important component of the process of photosynthesis. Most leaf nitrogen is bound in the proteins of the Calvin cycle and thylakoids. Because of this bondage, the N content is related to the photosynthetic capacity, and in general, in the thylakoid, it is proportional to the chlorophyll content [43]. The increased allocation of N to photosynthetic machinery can increase photosynthetic nitrogen use efficiency (PNUE) [42].

The processing and transportation of energy and 101 chemical compounds are very complex processes in vascular plants. In general, carbon is assimilated, and carbohydrates are synthesized in leaves and other photosynthesizing tissues, and they can then be transported to the roots. However, roots capture water and basic micro- and macroelements, which are transported in opposite directions. Nevertheless, these two processes are not entirely independent [44,45]. Moreover, despite the great degree of integrity of the plant’s body and efficient transportation systems, each plant shoot, root, or even leaf can have a great degree of independence [41,46,47]. A plant’s body can be divided into metamers that form modules and branches and show differences in their demographic performance and physiological properties (reviewed in [41]). Reproductive output can also differ depending on the plant’s module. For example, individual flowers within one plant can differ in nectar production [48], and the protein content in pollen grains can differ depending on the side of the tree crown [49]. However, physiological integration of plant modules may regulate reproductive costs and, for example, regulate the allocation of resources to current fruit development and future growth or allow only selected fruit to mature on the whole-tree level [50]. Moreover, the development and reproductive performance of branches growing in more favorable conditions (for example, light conditions) takes place at the cost of branches of the same tree but growing in less favorable conditions [51]. However, many studies show that assimilates and growth regulators are transported a short distance and are provided mainly by leaves from the same node, axil, or branch. Sometimes fruit is provided by assimilates from leaves growing on other growth units, but at the early stages of their maturation and during the most intensive phase of their growth they are provided by leaves from the same module (reviewed in [41]).

We hypothesized that female shoots with generative organs removed would have a decreased reproductive effort in comparison to control shoots, which is visible on the level of physiological and morphological changes and reproduction at the expense of shoots and leaves in close proximity. Because males are expected to have a smaller reproductive effort than females, we hypothesized that bud removal makes female shoots ecophysiologically more similar to males. In particular, we presumed the following:

**Hypothesis 1:**  *Control female shoots (F_c_) have smaller concentrations of nitrogen, carbon, and carbohydrates than bud-removed (F_b_) and male (M) shoots as a response to their greater reproductive effort*.

**Hypothesis 2:**  *Bud-removed female shoots invest more resources in vegetative growth than control female shoots.*

**Hypothesis 3:**  *Control female shoots show features of leaf morphology that help compensate for reproductive effort more efficiently in comparison to bud-removed female and male shoots*.

**Hypothesis 4:**  *Bud-removed and control female shoots do not differ in phenolic compound and tannin concentrations, but they differ from male shoots*.

## 2. Results

**Figure 1:**
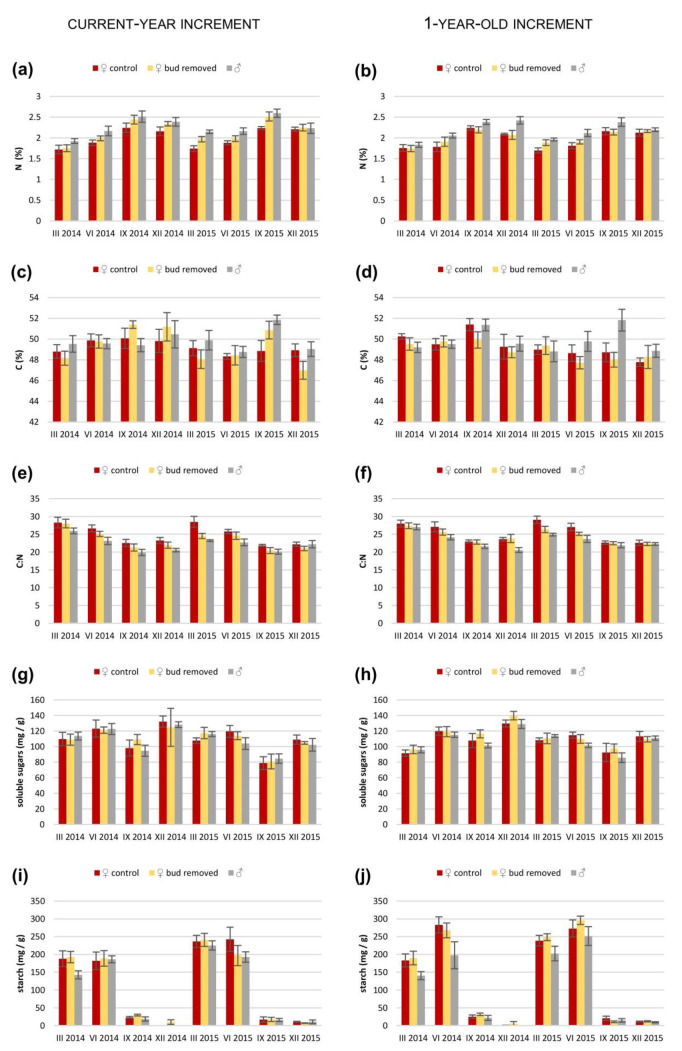
Mean values (±standard error) of concentration of (**a**,**b**) nitrogen; (**c**,**d**) carbon; (**e**,**f**) C:N ratio, and concentration of (**g**,**h**) soluble sugars and (**i**,**j**) starch in (**a**,**c**,**e**,**g**,**i**) current-year increments and (**b**,**d**,**f**,**h**,**j**) 1-year-old increments in control and bud-removed *Taxus baccata* female shoots and in male shoots measured in different seasons for two years.

In all three comparisons, most parameters differed depending on the season (Supplementary Table S2). The N concentration in current-year needles was different between the F_c_ and F_b_ shoots and between M and F_c_ shoots, but not between M and F_b_ shoots (Supplementary Table S2). It was significantly lower in the F_c_ shoots in comparison to the F_b_ shoots and M shoots (Supplementary Table S1; Figure 1). In 1-year-old needles, differences in N concentration values were present only between M and F_c_ shoots, and female shoots had a lower N concentration than male shoots (Supplementary Table S1; Figure 1). Moreover, the N concentration in both needle age classes changed with the season (Supplementary Table S2). However, the C concentration was not so volatile, but it was significantly different between males and both groups of female shoots in a group of 1-year-old needles (Supplementary Table S2), and it was lower in female shoots in both cases (Supplementary Table S1; Figure 1). The C:N ratio was different between the F_c_ and F_b_ shoots, as well as between M and F_c_ shoots in both current and 1-year-old needles (Supplementary Table S1; Figure 1). In both increments, it was lowest in males and highest in control females (Supplementary Table S1; Figure 1). Moreover, it changed with the season in both 1-year-old and current-year needles and in comparisons of sexes and both groups of female shoots (Supplementary Table S2).

The soluble sugar concentration was at the same level in all shoot types and in both sexes, and it differed across seasons (Supplementary Tables S1 and S2; Figure 1). Similarly, the starch concentration in current-year needles differed only in different seasons, but in 1-year-old needles, it was also different in comparisons of M to F_b_ shoots (Supplementary Table S2; Figure 1). The starch concentration was higher in F_b_ shoots than in M shoots (Supplementary Table S1). There were no differences in carbohydrate concentrations between the two types of female shoots, and this trend was present in both shoot ages.

**Figure 2:**
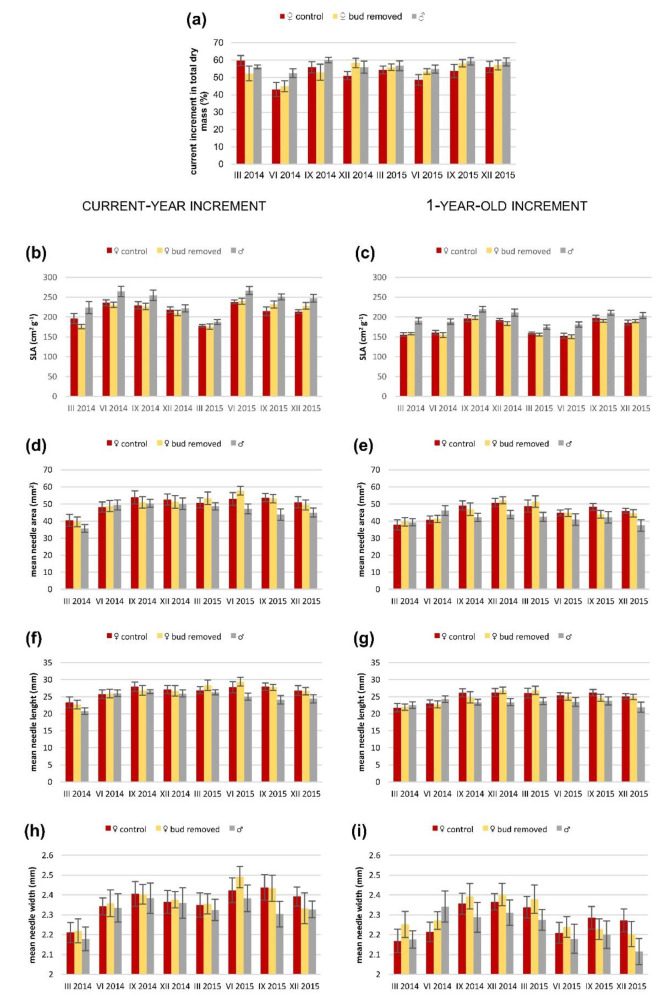
Mean values (±standard error) of (**a**) percentage of current-year increment in total dry mass of two-year increments; (**b**,**c**) specific leaf area; (**d**,**e**) mean needle area; (**f**,**g**) mean needle length and (**h**,**i**) mean needle width in (**b**,**d**,**f**,**h**) current-year increments; and (**c**,**e**,**g**,**i**) 1-year-old increments in control and bud-removed *Taxus baccata* shoots of females and males in different seasons for two years.

The percentage of current-year biomass increments in total two-year increments differed between M and F_c_ shoots but not between M and F_b_ shoots (Supplementary Table S2), and it was lower in F_c_ shoots (Supplementary Table S1). Moreover, it differed across seasons (Supplementary Tables S1 and S2; Figure 2).

The specific leaf area (SLA) values depended on the sex of the plants. Both groups of female shoots were different from male shoots in current and 1-year-old shoots (Supplementary Table S2), and females had smaller SLA values (Supplementary Table S1). A significant interaction was observed between season and sex in comparison to M and F_b_ 1-year-old shoots (Supplementary Table S2). However, no differences were detected between the F_c_ and F_b_ shoots (Figure 2; Supplementary Tables S1 and S2).

Parameters connected with leaf morphology were not different between sexes or between groups of female shoots. However, in 1-year-old needles, these parameters (mean needle: area, length, and width) were significantly different depending on season in all three comparisons (F_c_ vs. F_b_, M vs. F_c_, M vs. F_b_). Significant interactions between season and sex were observed between M and F_c_ shoots for mean needle area, length, and width in current-year needles. This interaction was not observed in comparisons between M and F_b_ shoots (Figure 2; Supplementary Tables S1 and S2).

**Figure 3:**
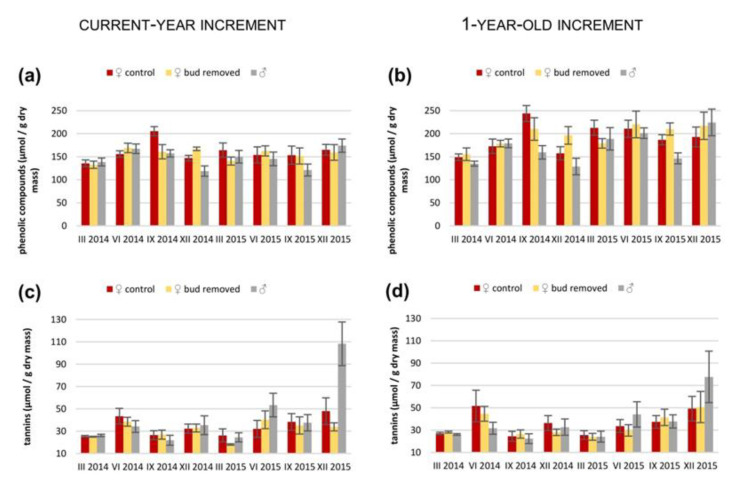
Mean values (±standard error) of (**a**,**b**) concentrations of phenolic compounds and (**c**,**d**) tannins in (**a**,**c**) current-year increments and (**b**,**d**) 1-year-old increments in control and bud-removed *Taxus baccata* shoots of females and in male shoots measured in different seasons for two years.

Concentrations of phenolic compounds from 1-year-old, but not current-year needles, were significantly different between sexes (Supplementary Table S2), and it was higher in female shoots than male shoots (Supplementary Table S1). It was not different between groups of female shoots in both current and 1-year-old needles, similar to the concentration of tannins (Supplementary Table S2). However, a significant interaction between season and sex was observed for current-year needles in tannin concentration, but only between M and F_b_ shoots (Supplementary Table S2; Figure 3). The concentration of tannins was also different depending on the season in both groups of needle ages and in all three comparisons (Supplementary Table S2).

## 3. Discussion

The reproductive effort of females relates to numerous consequences, including resource allocation and energy requirements (reviewed in [2,52,53]). These specific demands are not limited to the time of flowering but are present throughout the process of seed maturation. This forces females to adjust their metabolism to those demands. Some of those adjustments can be genetically based and some of them may be a response to current conditions.

### 3.1. Nitrogen

Nitrogen is one of the main elements related to the seed production of yew [54]. It is a component of proteins and nucleic acids. However, to the greatest extent, the development of seeds requires carbon and energy, predominantly obtained from leaves near developing seeds [37,38,39]. Moreover, obtaining energy depends on the allocation of nitrogen to the photosynthetic machinery, as it is used to the greatest extent to build Calvin cycle proteins and thylakoids [43]. In our experiment, nitrogen was allocated by females to generative organs rather than to photosynthetic machinery in leaves from proximity, as leaves from control shoots were visibly depleted of this element. Reproductive allocation relates to N depletion in nearby tissues, and the N concentration is greater in stems that do not bear fruit [50]. Differences observed in bud- and flower-removed experiments show that generative organ removal causes an N increase [55] in vegetative female tissues of dioecious species (but it is the opposite for males) [29]. N depletion in reproductive shoots confirms our Hypothesis 1, and it is a common allocation strategy in plants during seed development.

**Figure 4:**
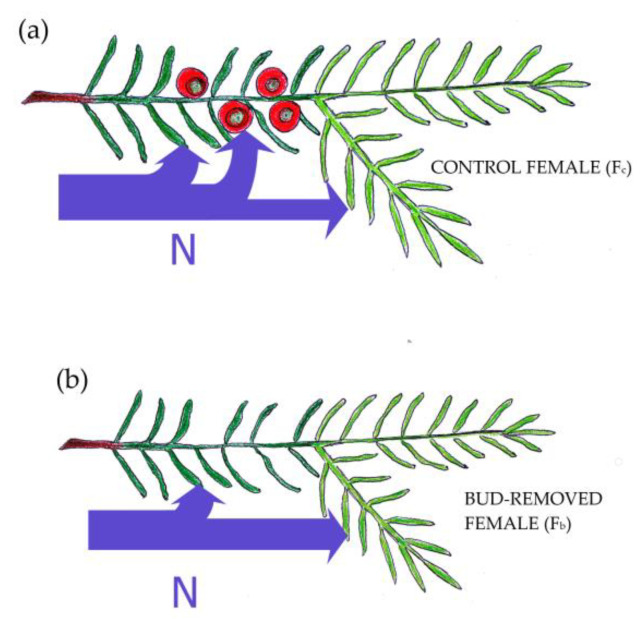
Diagram showing the flow of nitrogen, and possibly other elements, in *Taxus baccata* female shoots, depending on their reproductive efforts. N is transported to 1-year-old needles (dark green) and current-year needles (light green). (**a**) In the control female N is detained in developing arils, while (**b**) in the bud-removed female more N is available for developing, current-year needles.

The N concentration in female foliage increases with distance from reproductive structures and can have season-long effects [56]. Moreover, we observed that N for developing arils in *T*. *baccata* is obtained at the expense of current needles rather than 1-year-old needles. Arils are developed in 1-year increments, and resource transportation from roots leads from previous to current-year increments. Therefore, we presume that N is not directly transported from the current-year’s increment to developing arils. Instead, it is transported in smaller amounts through 1-year-old to current increments in control shoots because it is detained in arils (Figure 4). Moreover, the C:N ratio was higher in F_c_ shoots in comparison to F_b_ shoots of both needle ages. This shows that developing arils not only limit N transportation in current-year increments but also disrupt the C:N balance in 1-year-old increments, possibly also by increasing C uptake.

In addition, sex-related differences in N concentration, as well as C:N ratio, appeared only in relation of F_c_ shoots to M shoots, and they were absent between F_b_ and M shoots. A lower level of nitrogen concentration in the shoots and leaves of females was previously observed in other species [29,34,57,58]. However, differences between sexes are sometimes opposite [32] or absent [27]. Nevertheless, previous studies on *T*. *baccata* revealed that the nitrogen concentration in the needles of females producing seeds was lower in comparison to males [6]. Most of these results (including the present studies) are consistent with Jing and Coley’s [59] hypothesis of a lower N level and a higher C level in females of dioecious plant species. However, our present results indicate that these differences are directly related to different reproductive efforts and requirements. It has been suggested that a lower N concentration in females of dioecious plants in comparison to males is the consequence of reproductive effort and requirements for seed and fruit production (e.g., [34,57,58]). Higher N concentrations in M shoots compared to F_c_ shoots and the lack of differences between M shoots and F_b_ shoots are consistent with our previous assumptions (Hypothesis 1).

Our results showed that higher N concentrations in male leaves do not necessarily relate to greater demands for N to produce pollen grains; rather, differences between sexes are caused by greater N demand and usage for the development of seeds and arils. This means that female leaves are depleted of N and not that males increase the N accumulation in comparison to females. This can be presumed from the fact that female deprivation of generative structures made them statistically indistinguishable from males in N concentration. Although plants have developed a mechanism of element reabsorption that can limit nitrogen loss to generative organs, it can be applied to unused flower structures [60], such as unfertilized ovules, but not seeds or pollen [42]. Additionally, Nowak et al. [61] found that female yews have greater belowground allocation than males, but males have a greater specific root area, which allows them to have more efficient resource uptake. This mechanism can compensate for N use to produce pollen, as, despite reproductive effort, male shoots did not differ from non-reproducing female shoots.

Moreover, N patterns of investment in various functions can differ depending on the sex morph [62]. Therefore, bud-removed females may invest excess N in photosynthetic machinery to maintain a higher photosynthetic capacity and males may invest in nitrogen-rich pollen grains. In studies on *Siparuna grandiflora*, Nicotra [29] concluded that females use additional N to increase photosynthetic nitrogen use efficiency, while males probably store it for future reproduction. Previous studies on *T*. *baccata* [63] showed that male and female plants do not differ in photochemical capacity, even under poor nitrogen-limited conditions. Moreover, McDowell et al. [56] showed that no reduction in foliar photosynthesis was present in *Pseudotsuga menziesii*, despite depletion of foliar nitrogen. The authors hypothesized that cone development stimulates foliar photosynthesis in close proximity, which counteracts N deficiency in needles. A similar phenomenon can be responsible for the same photochemical capacity of the sexes in yew [63], despite lower nitrogen levels in reproductive female shoots compared to M and F_b_ shoots.

However, our results can explain why female specimens often have a lower growth rate in comparison with males in dioecious species [2,64], including yews [65]. The annual growth of shoots is correlated over time with the growth and development of seeds and arils, which seem to be a very important N sink. This process limits the N content in current-year shoots and, consequently, may limit the growth of females.

### 3.2. Carbon and Carbohydrates

Inconsistent with our Hypothesis 1, carbon accumulation remained unchanged in female shoots, regardless of the presence or removal of generative buds. This shows that generative organs are not strictly dependent on nearby leaves for carbon supplementation. Arils may supply themselves with carbon, at least during the initial period of development, because, while they are green, they can contribute to their carbon requirements [63]. Moreover, a period of intensive supplementation with carbon can be a very short stage in seed development. McDowell et al. [56] discovered that, in *Pseudotsuga menziesii,* needles in proximity to the generative organs had greater photosynthetic rates only during the initial period after the emergence of cones from the buds, and female cones started to photosynthesize one week after bud break. Additionally, the C concentration per unit of dry mass of cones remained relatively constant during cone maturation [56]. *Taxus baccata* female strobili open in March and the initial period of cone development occurs afterward. However, it is unlikely that cones can supply themselves with all C required for their development, as McDowell et al. [56] showed that, in *P*. *menziesii*, only 6% of C comes from the cones’ own photosynthesis. Moreover, it is unlikely that arils were supplemented with carbon originating from distant shoots, as many studies have shown that C for developing seeds in mature tree species comes from the same module (reviewed in [41]). However, equal C concentrations in the control and bud-removed shoots can also relate to changes in photosynthesis, as the removal of sink structures can lead to lower photosynthetic rates (reviewed in [41]), and generative shoots may compensate for their reproductive effort by more intensive carbon gain, which is not accumulated in leaves but transported to arils. It is probable, as numerous studies have shown, that a strong, developing sink is often related to an increase in photosynthesis in subtending or close leaves [66,67,68,69], but it is not always unambiguous [70]. Detailed measurements of photosynthesis should be applied to control and bud-removed shoots of *T*. *baccata* to determine if differences in carbon assimilation are responsible for the lack of differences in C concentrations between female shoot treatments.

However, between-sex differences in C concentration in our current studies are inconsistent with previous research, as Nowak-Dyjeta et al. [6] detected no differences between male and female *T*. *baccata* in C concentration, and Rabska et al. [57] observed higher C concentrations in females than males of *Juniperus communis*. In the current studies, a higher C concentration in male than female shoots of *T*. *baccata* in 1-year-old needles can relate to female preparation to support developing seeds (which appears in 1-year-old increments). However, it seems that this mechanism is independent of current reproductive effort.

Similar patterns can be expected for the production and allocation of carbohydrates, as they are the main carbon reservoirs prepared for present or future use in plants [71]. Our results for carbohydrates were inconsistent with our Hypothesis 1, as we observed only seasonal changes in soluble sugars and starch concentrations and differences in starch in 1-year-old increments between M and F_b_ shoots. Soluble sugars are used mostly immediately after synthesis, in contrast to starch [71], and differences between M and F_b_ with a lack of differences between M and F_c_ shoots can indicate a slightly greater accumulation of energy as starch because of suppressed reproduction in females. However, the difference was significant only in comparison to males. Nevertheless, previous studies on *T*. *baccata* and *J*. *communis* have shown greater starch accumulation in females than in males [57,72]. Now, we can conclude that this pattern exists independent of reproductive effort, but it can be intensified by the suppression of female reproduction. Avila-Sakar et al. [73] showed that resources not used in fruit production can be used in next flower bud production and growth. Previous studies have shown, however, that the lack of influence of reproductive effort on starch accumulation is not a universal mechanism, and it can increase in treated shoots [19]. However, studies conducted on *Aesculus californica* have shown that the total non-structural carbohydrate concentration is not different between shoot-bearing and non-bearing fruit [50]. The author explains this as the result of carbohydrate transportation from roots and stems, which can be true in the current studies.

### 3.3. Vegetative Growth

Nevertheless, many studies, not only concerning dioecious species, show that a trade-off exists between generative and vegetative growth and reproduction (e.g., [50,74,75,76]). The removal of generative buds, flowers, or fruit, in most cases, stimulates vegetative growth [1], especially in females [28], and plants with removed buds can increase their dry weight in periods when control plants cannot [55]. However, in our studies, strobili bud removal did not produce such an effect. The lack of differences in the increase in shoot growth between female shoots is in contrast to our expectations (Hypothesis 2). There are many explanations, but the most important one seems to be that the observations lasted only two years, and the differences between the sexes in height or radial growth accumulate over many years in woody species [77]. However, other explanations are also possible. Milla et al. [32] found that there was no trade-off between fruit load and investment in current-year leaves in female *Pistacia lentiscus*. Such unexpected results can be explained by resource translocation [4]. However, resource translocation is a very limited process, and module independence is the dominant scheme in plants [46]. Moreover, compensatory mechanisms of allocation from non-reproductive shoots [52] are not justified in plants, where most shoots bear generative organs [32].

However, observed differences between M and F_c_ shoot increments showed that the reproductive effort of females can explain the smaller vegetative biomass increments (Supplementary Tables S1 and S2). Moreover, intermediate values of F_b_ shoots for this parameter showed that this process is not strictly dependent on plant sex and that different factors can affect it. F_b_ shoots were indirectly connected with F_c_ shoots, and they could affect each other to some degree, unlike if they were on separate plants.

In *Taxus baccata*, female generative organs develop in 1-year increments, and other studies concerning dioecious *Pistacia lentiscus* have shown that resources for current-year reproduction are sourced from old branch stores and not from current-year vegetative increments [78]. Therefore, current-year increments are sources of nutrients and energy for next season reproduction [74] and young increments are rather a sink of assimilates from older parts of the plant than sources of them (reviewed in [41]). Because of reproductive investment in 1-year-old neighboring increments in F_c_ shoots, a smaller resource amount was available for current-year growth. This was also confirmed by the results of the N concentration described above.

Many studies have shown a decrease in female size and inter alia tree ring growth after the start of reproduction [5,77,79], and some authors presume that reproductive costs can be compensated by resources from adjacent shoots [52,79]. Although many authors have indicated a great degree of module independence, the sectorial structure of plants connects leaves with shoots and roots [41,46]. The architectural location of specific fragments of organs plays an important role in the degree of their physiological connection, indicating that a particular leaf can support a particular non-photosynthesizing organ [80,81,82,83], but those relationships are not entirely exclusive [38]. Therefore, we suspect that shoots that bear arils do not differ in their biomass from bud-removed shoots because they can compensate for resource loss at the expense of the increase in biomass of the main trunk, especially tree ring growth, contributing less resources than non-reproductive adjacent shoots to non-photosynthesizing vegetative tissues.

However, because shoots with reproductive effort can show more intensive photosynthesis [41,56], equal biomass in F_c_ and F_b_ shoots may be a manifestation of increased resource uptake and greater energy assimilation during the development of generative buds and arils. Although a lower photosynthetic efficiency can be observed in fruiting female shoots than in non-fruiting female shoots [79], higher light-saturated photosynthetic rates can also be observed in fruiting females in comparison to unpollinated females [27]. Moreover, in our studies, results describing plant growth are consistent with those for C concentration, showing no differences between female shoots despite variations in reproductive effort. This indicates that, in *T*. *baccata*, increased photosynthesis in the reproductive shoots of fully mature females is probable. However, studies conducted on the rooted shoots of yew show that photochemical capacity is not different between sexes and does not compensate for females’ greater reproductive effort [63]. However, there is proof that vegetative and generative growth are not always opposite processes, and that plants with and without inflorescences can have a similar rate of canopy expansion [84]. In our studies, F_c_ shoots had a lower increase in growth than M shoots despite the potentially similar photochemical capacity of the sexes [63], which indicates that the trade-off between vegetative and generative growth is more pronounced in females than males because of their greater reproductive effort.

### 3.4. Needle Morphology

Specific leaf area (SLA) is a parameter that describes the trade-off between the accumulation of biomass in leaves and investment in photosynthetic areas [85]. We hypothesized that in our studies, F_c_ shoots compensated for greater reproductive effort by increasing their SLA in comparison to F_b_ and M shoots. The increased photosynthetic area can result in increased C assimilation, resulting in dry mass investment. However, we did not confirm our Hypothesis 3, as SLA was higher in males and this trend was independent of female shoots’ reproductive effort. We found that SLA was not strictly dependent on local current reproductive effort but related to the interaction between sex and local light conditions. Nevertheless, previous studies on *T*. *baccata* revealed no differences in SLA between sexes [6], which confirms that not only reproductive effort and sex, but also other factors, can have an influential impact on the variability of this parameter in yew.

The parameters of needle morphology, similar to SLA, can relate to compensation mechanisms in females. Numerous studies have shown that females have a greater leaf area (e.g., [86,87]), which has also been shown in *T*. *baccata* [6]. The lack of differences between both groups of female shoots shows that, in *T*. *baccata*, the parameters of needle morphology are not strictly connected with current local reproductive effort, and this is inconsistent with our Hypothesis 3. Perhaps the time of bud removal should be longer to have a visible influence on needle morphology, but needle morphology in yew may not respond to local current reproductive effort, and its liability is not connected with an investment in reproduction. However, a reduction in flower density leads to an increase in leaf area [88]. Other studies have shown a lack of differences between control and threatened plants [89,90], indicating that leaf area does not always respond to changes in biomass allocation. Pettigrew et al. [89] suggested that resources not used in generative organ development can be allocated to stems and roots instead of leaves. It is possible that *T*. *baccata* also allocates saved resources to different sink structures.

### 3.5. Phenols and Tannins

Previous studies on *T*. *baccata* have shown that phenolic compound concentration is higher in females, and this phenomenon was also observed before sex maturation, which shows that it is independent of the allocation for reproduction [72]. Our results confirm this trend, as well as our Hypothesis 4, because differences were observed between sexes in old, fully developed needles, and at the same time, this trend remained constant, independent of female shoots’ reproductive effort. Therefore, reproductive effort does not influence phenolic compound concentration, and this confirms its constitutive character. However, a trade-off between reproduction and other functions exists in dioecious species, such as fruiting females of *Salix rigida* (but not reproductive males), which contain fewer secondary metabolites and produce fewer generative buds and shorter shoots than non-fruiting females [3]. The lack of differences observed in our studies can indicate that, in females of *T*. *baccata*, in contrast to *S*. *rigida*, secondary metabolite production is not dependent on allocation for reproduction.

Moreover, in our studies, the tannin concentration depends on the season in relation to sex but only in comparison to M and F_b_ shoots. This indicates the influence of differences connected with the reproductive roles of sexes and local spatial segregation in relation to seasonal changes on the level of tannin concentration. Condensed tannins play antioxidative roles in response to drought [91], and female *T*. *baccata* trees occur on wetter microsites more often than males [92]. Therefore, we suspect that higher periodical tannin concentrations relate to water shortages and are especially visible in males (Figure 2).

Phenolic compounds are responsible for plants’ response to stress [93,94], and many studies have shown that females of dioecious species have a greater phenolic concentration [34,57]. As a different strategy for resource allocation, females invest in defense and males in growth, especially in unfavorable conditions [95]. In addition to phenolics, tannins take part in stress response, and levels of both kinds of compounds constitute defensive strategies (reviewed in [96]). In dioecious plants, females invest more, as was shown in *Acer negundo* [59] and *S*. *rigida* [3], but not in *Spondias purpurea* [86]. Moreover, a trade-off between growth and defense exists for females (but not males), and females with greater biomass can have greater phenolic and smaller tannin concentrations [97]. However, similar to studies conducted on *Baccharis dracunculifolia* [98,99] and *Populus tremula* [100], we did not note differences in tannin concentration between sexes. This shows that the level of total phenolic compounds, rather than the level of tannins, is related to plant sex in *Taxus baccata*, constituting a sex-related strategy. Moreover, this trend indicates that the growth–defense balance can change between sexes, but it can also depend on specific compounds.

The main postulated weakness of bud removal experiments is that the process of bud removal causes injury and, as a result, the plant activates mechanisms of wounding response [18]. Phenolic compounds are synthesized as a response to injury and pathogens in tree species [94,101]. The tannin concentration was found to increase in response to grazing [102,103,104,105], (reviewed in [106]), but tannins can be present constantly, and only their level changes as a response to stress [104]. Moreover, phenolic compounds are produced locally in the branch that has been damaged [107], but tannin production in leaves competes with carbohydrate sinks, such as roots [104]. Therefore, we concluded that bud removal in our experiment did not have a significant influence on stress reaction, as both phenolic compounds and tannin concentrations were equal in statistical analysis in both groups of female shoots.

## 4. Materials and Methods

### 4.1. Plant Material and Experiment

*Taxus baccata* L. is a coniferous species from the Taxaceae family. It is an evergreen dioecious plant growing in Europe, north Africa, and west Asia. Mature individuals of *T*. *baccata* growing in the Arboretum of the Institute of Dendrology Polish Academy of Sciences in Kórnik, Poland, were used in the experiment. Individuals were about 10 m high, and they were about 65 years old [108]. The experiment was introduced in 2013, one month after pollination, when all generative organs were removed from the designated shoots of female individuals. At the same time, control shoots without any interference were designated for the same female plants and male individuals. In the results, three types of shoots were analyzed: control female (F_c_), bud-removed female (F_b_), and male (M). Branches selected for the experiment were placed at the same height and at the same position in relation to the sun. Bud removal was repeated each year in the spring, one month after pollination.

Beginning in March 2014, in each season, the chemical compositions of vegetative shoots, biomass, and needle morphology were measured (Table 1). Measurements were conducted over two years in March, June, September, and December. Those months were chosen because they represent specific phenological phases: strobili production in spring, intensive vegetative growth in summer, biomass allocation to roots in autumn, and dormancy in winter. At each measurement time, the features of shoots from 10 female (F) and 10 male (M) individuals (three shots per individual) were measured. Shoots were additionally divided into current-year shoots and 1-year-old shoots. As a result, 120 shoots were analyzed (10 individuals × 2 sexes × 3 shoots × 2 increments). For elements and chemical compound analysis, needles (divided into current and 1-year-old) were separated from shoots, dried at 65 °C for 72 h, and ground to a fine powder with a Mikro-Feinmühle Culatti mill (IKA Labortechnik, Staufen, Germany).

### 4.2. Elements

Carbon and nitrogen concentrations were determined in the needle powder using an Elemental Combustion System CHNS-O Analyzer (Perkin Elmer; Costech Analytical Technologies Inc., Valencia, CA, USA). The percentages of N and C were determined and additionally used for the C:N ratio calculation.

### 4.3. Carbohydrates

Soluble sugars and starch concentrations were determined using the modified methods of Haissig and Dickson [109] and Hansen and Møller [110] according to a description published in Oleksyn et al. [111]. From powdered tissues, carbohydrates were extracted in a mixture of methanol:chloroform:water in the ratio of 12:5:3, respectively. The anthrone reagent was used to colorimetrically determine the soluble sugar concentrations in the extracts. Measurements were conducted at a wavelength of 625 nm. The starch in the tissue residue was converted to glucose with amyloglucosidase. It was then incubated for 30 min with peroxidase-glucose oxidase-odianisidine dihydrochloride at 25 °C. After incubation, absorbance was measured at 450 nm. The concentrations of soluble sugars and starch were expressed as a percentage of glucose per g of needle dry mass. Soluble sugar concentrations were calculated from linear regression using standard glucose solutions.

### 4.4. Phenols and Tannins

The total phenolic compound concentration (TPhC) was measured using 0.1 g of needle powder. It was boiled for 15 min in 95% ethanol and 10 min in 80% ethanol. The Folin–Ciocalteu Phenol Reagent (Sigma F-9252) was used, and the total phenol content was measured spectrophotometrically with a 660 nm wavelength according to Johnson and Schaal [112] and modified by Singleton and Rossi [113]. The results were expressed as µmol of chlorogenic acid per g of dry mass.

Condensed tannins were extracted using a method proposed by Price et al. [114] and 0.025 g of powdered needles were used. Absolute methanol was added to the samples and kept at room temperature for 20 min. To perform the color reaction, 0.5% vanillin solution in 4% HCl solution in absolute methanol was used, and the wavelength for absorption measurements was 500 nm. The results were expressed as µmol of catechin per g of dry mass, as catechin was used as a standard.

### 4.5. Morphology

Morphological measurements were conducted on fresh needles. 30 needles from current and 30 needles from 1-year-old shoots were separated from shoots and scanned in an Epson Perfection V700 Photo scanner. WinSeedle (Regent Inc. Quebec City, QC, Canada) image analysis systems were used, and the following parameters were obtained: mean needle area, mean needle length, and mean needle width. Additionally, needles and shoots were dried (as described above), and the dry mass of current and 1-year-old increments was measured. Afterwards, the percentage of current-year increment mass in total two-year increments of biomass was calculated. The total needle area per mass ratio was calculated to obtain a specific leaf area (SLA) value.

### 4.6. Statistical Analysis

Statistical analyses were conducted using JMP^®^ 15.2.0 Pro software (SAS Institute Inc., Cary, NC, USA, 1989–2019). Data obtained in percentages were modified with Bliss correction [115]. Prior to the statistical analysis, outliers were identified. Methods proposed by Dean et al. [116] were used and studentized residual plots and residual normal quantile plots were analyzed. Moreover, quantile range (0.1–3 Q) outliers were identified. Comparisons were divided into three groups and evaluated separately: F_c_ shoots vs. F_b_ shoots, F_b_ shoots vs. M shoots, and F_c_ shoots vs. M shoots. For two-way analysis of variance (ANOVA), sex (or bud removal in comparison of female shoots) and season were used as sources of fixed effects. Individual and year of measurements nested in individuals were used as random effects. When variability occurred, a Student’s t-test was used to compare the mean values, and for multiple comparisons (interactions between season and sex or bud removal), Tukey’s HSD test was used. Data are presented as the mean ± standard error (SE). Differences were considered significant at *p* < 0.05.

## 5. Conclusions

Females allocate nitrogen to developing seeds rather than to photosynthetic machinery in the leaves. N transportation to seeds limits its delivery to current-year increments. At the same time, there being no differences in N content between F_b_ female and M shoots shows that, as observed in many dioecious species, greater N content in males is caused by usage of this element in female reproduction rather than increased accumulation in male tissues. This pattern of allocation is present even though nitrogen allocation to photosynthetic machinery could increase carbon gain, which builds energy carriers and is the main element required for developing seeds. However, there were no differences in carbon and carbohydrate concentrations between the F_c_ and F_b_ shoots, suggesting two probable scenarios. The first is that generative organs are supplemented in carbon and carbohydrates at the expense of the main trunk (e.g., tree ring growth) rather than shoots in proximity. The second is that manipulation of reproductive effort can change photosynthetic efficiency, which requires further research. These scenarios are likely, as no differences were observed between the F_c_ and F_b_ shoots in vegetative growth and leaf morphology. Moreover, the production of generative organs in females does not come at the cost of defensive metabolite synthesis, and differences between males and females in phenols concentration seem to appear independent of reproductive effort.

The study showed that bud removal in *T. baccata* branches of females make them biochemically more similar to males. It reveals the significance of reproductive effort and reproductive allocation on females biochemistry, which can consequently influence their growth and performance.

## Figures and Tables

**Table 1:** The scheme of sampling of *Taxus baccata* shoots during 2-year bud-removal experiment and the division of increments on current and 1-year-old shoots at each sampling date.

		Year in Which the Increment Grew			
		2012	2013	2014	2015
sampling date	III 2014	1-year-old	current-year		
	VI 2014		1-year-old	current-year	
	IX 2014		1-year-old	current-year	
	XII 2014		1-year-old	current-year	
	III 2014		1-year-old	current-year	
	VI 2015			1-year-old	current-year
	IX 2015			1-year-old	current-year
	XII 2015			1-year-old	current-year

## Data Availability

Not applicable.

## Supplementary Materials

The following supporting information can be downloaded at: https://www.mdpi.com/article/10.3390/ijms232214225/s1.
