# Supplementary material for: Consequences of the Reproductive Effort of Dioecious Taxus baccata L. Females in a Generative Bud Removal Experiment—Important Role of Nitrogen in Female Reproduction
Source: Int J Mol Sci. 2022 Nov 17;23(22):14225. doi: 10.3390/ijms232214225 (PMC9695432; doi:10.3390/ijms232214225)
Supplement: Supplementary file 1 [file ijms-23-14225-s001.zip › Table S1.pdf]

Supplementary Table S1. Values of mean and standard error (SE) of nitrogen and carbon concentration, C:N ratio, soluble sugar and starch concentration, percent of mass of current increment in total two-year increment mass, specific leaf area (SLA), mean leaf area, mean leaf length, mean leaf width, and phenolic compound and tannin concentration for *Taxus baccata* male and two female shoots (control and bud-removed) measured on subsequent dates. Shoots of different ages (current and 1-year-old increments) were analysed separately.

		control female		bud- removed female		male	
	date	mean	SE	mean	SE	mean	SE
N current year (%)	III 2014	1.72	0.10	1.75	0.08	1.92	0.06
	VI 2014	1.88	0.07	1.99	0.06	2.17	0.12
	IX 2014	2.24	0.11	2.44	0.11	2.51	0.14
	XII 2014	2.16	0.10	2.34	0.05	2.39	0.10
	III 2015	1.74	0.07	1.97	0.06	2.15	0.04
	VI 2015	1.88	0.05	1.98	0.07	2.16	0.08
	IX 2015	2.24	0.04	2.52	0.11	2.60	0.10
	XII 2015	2.21	0.05	2.25	0.08	2.23	0.13
	total	1.99	0.04	2.12	0.05	2.24	0.04
N previous year (%)	III 2014	1.76	0.08	1.74	0.07	1.84	0.06
	VI 2014	1.78	0.12	1.91	0.11	2.06	0.06
	IX 2014	2.24	0.05	2.19	0.07	2.38	0.06
	XII 2014	2.09	0.03	2.07	0.11	2.42	0.09
	III 2015	1.70	0.06	1.88	0.07	1.96	0.04
	VI 2015	1.81	0.08	1.90	0.05	2.12	0.09
	IX 2015	2.16	0.09	2.14	0.07	2.38	0.10
	XII 2015	2.13	0.08	2.17	0.04	2.20	0.04
	total	1.94	0.04	1.98	0.03	2.14	0.04
C current year (%)	III 2014	48.80	0.66	48.16	0.68	49.54	0.80
	VI 2014	49.87	0.62	49.77	0.64	49.57	0.50
	IX 2014	50.07	0.98	51.40	0.36	49.41	0.64
	XII 2014	49.82	1.13	51.19	1.37	50.47	1.32
	III 2015	49.12	0.74	48.06	0.90	49.89	0.95
	VI 2015	48.33	0.28	48.43	0.94	48.76	0.54
	IX 2015	48.86	1.01	50.87	0.85	51.86	0.46
	XII 2015	48.92	0.62	46.98	0.86	49.03	0.70
	total	49.19	0.27	49.27	0.35	49.78	0.29
C previous year (%)	III 2014	50.26	0.26	49.52	0.60	49.20	0.50
	VI 2014	49.50	0.56	49.77	0.54	49.51	0.40
	IX 2014	51.40	0.58	49.91	0.78	51.37	0.56
	XII 2014	49.26	1.19	48.73	0.53	49.55	0.73
	III 2015	48.98	0.47	49.37	0.85	48.81	1.00
	VI 2015	48.64	0.80	47.72	0.60	49.78	0.97
	IX 2015	48.72	0.90	48.00	0.72	51.83	1.05
	XII 2015	47.77	0.41	48.28	1.12	48.87	0.64

	total	49.37	0.26	48.94	0.27	49.81	0.28
C:N current year	III 2014	28.27	1.48	28.00	1.22	25.94	0.81
	VI 2014	26.63	0.96	25.09	0.72	23.12	1.03
	IX 2014	22.56	1.02	21.27	1.00	19.89	0.87
	XII 2014	23.25	0.86	21.94	0.88	20.55	0.48
	III 2015	28.49	1.52	24.51	0.72	23.23	0.25
	VI 2015	25.75	0.63	24.59	1.04	22.71	0.95
	IX 2015	21.85	0.28	20.39	0.84	20.11	0.72
	XII 2015	22.19	0.60	21.01	0.64	22.23	1.02
	total	25.07	0.52	23.71	0.50	22.51	0.40
C:N previous year	III 2014	28.00	0.99	27.39	0.79	27.03	0.79
	VI 2014	27.07	1.38	25.60	0.87	24.17	0.76
	IX 2014	23.00	0.42	22.83	0.59	21.62	0.61
	XII 2014	23.59	0.53	23.82	1.17	20.56	0.68
	III 2015	29.05	1.04	26.34	0.87	24.90	0.37
	VI 2015	27.04	1.06	25.13	0.44	23.68	0.99
	IX 2015	22.63	0.49	22.50	0.45	21.91	0.73
	XII 2015	22.61	0.76	22.31	0.47	22.27	0.38
	total	25.45	0.46	24.59	0.36	23.56	0.38
soluble sugars current year (mg/g^{-1})	III 2014	109.61	8.61	108.93	7.22	113.56	5.11
	VI 2014	123.08	11.01	121.20	4.01	122.70	7.01
	IX 2014	98.07	10.29	109.08	6.57	94.65	7.06
	XII 2014	132.10	7.18	124.77	24.48	128.25	3.71
	III 2015	107.72	3.51	117.37	7.27	116.17	3.22
	VI 2015	119.58	7.64	113.98	5.04	103.92	7.42
	IX 2015	78.83	8.26	80.88	9.54	84.63	6.09
	XII 2015	108.88	5.89	104.73	1.69	102.32	8.08
	total	109.73	3.44	110.03	3.75	108.68	2.74
soluble sugars previous year (mg/g^{-1})	III 2014	91.56	4.11	96.38	5.42	95.85	3.96
	VI 2014	119.87	5.49	119.18	6.51	115.24	3.26
	IX 2014	107.65	9.11	116.42	5.19	101.48	3.16
	XII 2014	129.62	4.40	139.78	5.52	129.05	5.80
	III 2015	108.12	3.04	110.55	6.73	113.68	1.71
	VI 2015	114.68	3.93	109.77	5.60	101.63	2.91
	IX 2015	92.50	11.78	97.63	5.68	85.73	6.10
	XII 2015	113.23	6.38	109.50	3.51	110.82	2.71
	total	108.15	2.68	111.17	2.61	105.67	2.21
starch current year (mg/g^{-1})	III 2014	187.76	22.24	192.63	15.96	142.31	11.43
	VI 2014	181.78	24.98	188.75	22.03	186.35	9.80
	IX 2014	22.97	2.43	29.72	2.27	18.53	5.81
	XII 2014	0.00	0.00	9.90	6.32	0.00	0.00
	III 2015	236.50	17.31	240.67	18.32	225.00	12.93
	VI 2015	242.32	34.14	196.83	28.24	192.57	14.68
	IX 2015	17.17	7.53	17.65	6.03	15.87	4.30

	XII 2015	10.87	1.13	6.98	0.64	10.98	5.20
	total	118.21	15.37	116.72	14.25	102.29	12.67
starch previous year (mg/g^{-1})	III 2014	182.79	18.31	189.62	19.07	140.37	11.23
	VI 2014	282.90	22.86	267.77	20.89	197.62	37.64
	IX 2014	25.08	5.05	31.80	3.55	21.70	6.97
	XII 2014	0.63	0.56	5.88	5.88	0.00	0.00
	III 2015	238.85	14.77	248.53	9.80	202.35	20.38
	VI 2015	272.95	23.87	295.90	12.05	251.60	26.65
	IX 2015	20.80	6.13	10.80	2.52	14.63	4.80
	XII 2015	11.57	1.15	12.07	1.75	9.30	1.61
	total	133.55	16.87	137.17	17.04	107.44	14.41
current increment mass in totaled two-year increments mass (%)	III 2014	59.75	2.95	52.38	4.19	56.09	1.14
	VI 2014	43.14	4.05	45.01	3.14	52.45	2.52
	IX 2014	55.90	3.24	53.02	4.68	60.06	1.60
	XII 2014	51.08	2.40	58.41	2.73	55.96	3.56
	III 2015	54.36	2.29	55.88	1.87	56.76	2.82
	VI 2015	48.60	3.11	53.56	1.60	54.84	2.27
	IX 2015	53.69	3.80	58.30	2.17	59.36	2.11
	XII 2015	56.03	3.25	57.20	2.75	58.99	2.26
	total	52.82	1.20	54.23	1.12	56.81	0.85
SLA current year (cm^2 g^{-1})	III 2014	196.10	12.66	176.12	5.60	223.56	15.18
	VI 2014	235.64	8.00	230.77	6.35	264.48	13.42
	IX 2014	229.41	9.02	226.79	7.89	254.87	13.42
	XII 2014	218.45	7.37	210.35	7.03	221.93	9.13
	III 2015	178.00	3.03	176.24	6.77	187.52	6.28
	VI 2015	238.23	5.10	239.73	7.82	267.00	9.88
	IX 2015	214.75	10.97	231.89	8.93	251.04	7.59
	XII 2015	213.41	5.08	228.53	8.31	247.33	9.90
	total	215.12	3.59	215.05	3.79	240.77	4.76
SLA previous year (cm^2 g^{-1})	III 2014	155.85	5.02	158.29	2.97	190.51	7.86
	VI 2014	160.88	5.84	154.74	6.07	188.78	6.76
	IX 2014	196.89	8.85	198.26	4.68	219.55	7.22
	XII 2014	192.46	4.13	183.44	4.40	211.38	8.97
	III 2015	159.31	2.59	155.66	3.69	174.77	5.42
	VI 2015	153.01	6.63	150.41	4.55	181.27	6.30
	IX 2015	197.55	7.51	190.48	3.65	210.85	5.86
	XII 2015	185.86	6.39	189.98	3.82	203.82	7.30
	total	174.25	2.99	171.94	2.60	197.47	2.92
mean leaf area current year (mm^2)	III 2014	40.35	3.50	39.62	2.87	35.74	2.28
	VI 2014	48.25	2.93	48.86	3.20	49.41	2.96
	IX 2014	53.87	3.72	50.96	3.33	50.42	2.31
	XII 2014	52.51	3.21	51.25	3.66	50.12	3.30
	III 2015	50.73	2.87	53.34	3.60	48.69	2.14
	VI 2015	52.93	3.68	57.69	2.59	47.12	2.87

	IX 2015	53.66	2.38	53.12	2.38	43.82	3.40
	XII 2015	51.00	3.18	49.46	2.88	44.99	2.66
	total	50.41	1.18	50.45	1.18	46.29	1.07
mean leaf area previous year (mm^2)	III 2014	37.82	2.98	39.60	2.51	39.30	2.09
	VI 2014	40.86	2.23	41.35	2.11	46.19	2.78
	IX 2014	49.12	2.68	46.99	3.76	42.26	2.36
	XII 2014	50.78	2.36	52.14	1.94	43.85	2.49
	III 2015	48.81	3.44	51.36	3.34	42.51	2.58
	VI 2015	44.76	1.74	44.89	2.21	40.90	3.45
	IX 2015	48.36	2.10	44.07	2.27	42.22	3.28
	XII 2015	45.97	1.45	44.57	2.12	37.45	3.40
	total	45.81	0.95	45.63	1.00	41.89	0.99
mean leaf length current year (mm)	III 2014	23.34	1.56	22.67	1.28	20.79	0.97
	VI 2014	25.75	1.29	25.91	1.22	25.99	0.96
	IX 2014	27.90	1.42	26.87	1.43	26.48	0.58
	XII 2014	27.09	1.21	26.70	1.53	25.93	1.02
	III 2015	26.91	1.08	28.34	1.54	26.28	0.78
	VI 2015	27.75	1.62	29.38	1.29	25.00	1.00
	IX 2015	27.95	1.06	27.72	0.86	24.09	1.24
	XII 2015	26.78	1.44	26.61	1.11	24.38	1.17
	total	26.68	0.48	26.74	0.49	24.87	0.39
mean leaf length previous year (mm)	III 2014	21.78	1.24	21.95	0.95	22.51	0.99
	VI 2014	22.99	1.05	22.74	1.00	24.26	0.98
	IX 2014	26.12	1.19	24.78	1.68	23.40	0.82
	XII 2014	26.25	1.16	26.87	0.99	23.39	1.03
	III 2015	26.05	1.45	26.85	1.20	23.71	0.99
	VI 2015	25.35	0.84	25.04	1.07	23.44	1.30
	IX 2015	26.17	0.92	24.69	1.01	23.79	1.19
	XII 2015	25.08	0.76	24.92	0.80	21.94	1.46
	total	24.97	0.41	24.73	0.42	23.32	0.38
mean leaf width current year (mm)	III 2014	2.21	0.05	2.22	0.06	2.18	0.06
	VI 2014	2.34	0.04	2.36	0.07	2.34	0.07
	IX 2014	2.40	0.06	2.40	0.05	2.38	0.08
	XII 2014	2.36	0.06	2.38	0.04	2.36	0.08
	III 2015	2.35	0.06	2.36	0.05	2.32	0.05
	VI 2015	2.42	0.06	2.49	0.05	2.38	0.07
	IX 2015	2.44	0.06	2.43	0.07	2.30	0.06
	XII 2015	2.39	0.05	2.33	0.08	2.33	0.04
	total	2.37	0.02	2.37	0.02	2.32	0.02
mean leaf width previous year (mm)	III 2014	2.17	0.06	2.25	0.07	2.18	0.04
	VI 2014	2.21	0.05	2.27	0.04	2.34	0.08
	IX 2014	2.36	0.05	2.39	0.06	2.29	0.08
	XII 2014	2.37	0.04	2.40	0.06	2.31	0.06
	III 2015	2.34	0.05	2.38	0.07	2.27	0.05

	VI 2015	2.21	0.05	2.24	0.05	2.18	0.07
	IX 2015	2.29	0.06	2.23	0.05	2.20	0.07
	XII 2015	2.27	0.06	2.20	0.06	2.11	0.07
	total	2.28	0.02	2.30	0.02	2.24	0.02
phenolic compounds current year (μmol/ g^{-1} dry mass)	III 2014	135.62	7.43	132.47	7.80	138.68	8.55
	VI 2014	155.84	6.89	169.29	10.36	167.31	10.41
	IX 2014	205.38	9.95	160.73	15.53	157.45	7.89
	XII 2014	147.05	6.13	167.12	3.79	118.76	11.17
	III 2015	164.44	15.35	140.72	8.69	150.02	13.82
	VI 2015	153.87	17.37	162.74	10.97	145.37	14.95
	IX 2015	153.38	19.82	151.62	17.59	121.09	13.00
	XII 2015	165.03	11.54	159.43	17.04	174.19	14.40
	total	158.19	4.85	153.74	4.31	146.00	4.61
phenolic compounds previous year (μmol/ g^{-1} dry mass)	III 2014	148.86	7.26	155.47	13.62	135.06	5.44
	VI 2014	172.76	15.53	178.45	7.21	178.98	9.48
	IX 2014	243.86	17.24	210.35	24.46	159.57	14.58
	XII 2014	157.46	14.40	196.37	19.04	128.73	17.95
	III 2015	212.85	16.28	179.03	10.25	188.93	24.10
	VI 2015	210.69	18.68	220.25	28.91	201.34	11.41
	IX 2015	186.86	10.92	209.99	13.24	146.44	11.92
	XII 2015	192.90	21.50	217.19	29.52	224.37	28.92
	total	188.31	6.52	192.78	7.13	167.71	6.85
tannins current year (μmol/ g^{-1} dry mass)	III 2014	25.34	0.63	24.96	0.31	26.16	1.02
	VI 2014	43.39	6.99	38.47	3.98	34.18	5.26
	IX 2014	26.41	3.97	26.81	4.14	21.78	4.38
	XII 2014	32.19	4.05	32.82	3.51	35.30	8.41
	III 2015	26.10	5.81	18.08	0.66	24.32	4.08
	VI 2015	32.01	7.55	40.15	7.88	53.32	10.55
	IX 2015	38.20	7.42	35.01	7.66	37.48	7.34
	XII 2015	47.85	11.99	33.76	3.58	108.31	19.55
	total	33.27	2.33	30.67	1.76	41.34	4.57
tannins previous year (μmol/ g^{-1} dry mass)	III 2014	27.05	1.12	28.14	0.84	26.05	0.76
	VI 2014	51.54	14.26	44.60	6.69	31.59	5.38
	IX 2014	24.41	4.43	26.41	3.67	22.29	4.31
	XII 2014	36.25	6.70	28.01	2.96	32.60	7.27
	III 2015	25.56	3.86	23.87	3.06	24.11	4.84
	VI 2015	33.50	5.83	29.67	5.10	43.99	11.31
	IX 2015	37.31	5.69	41.35	7.29	37.75	5.85
	XII 2015	49.16	11.06	50.59	13.93	77.65	23.13
	total	34.94	2.68	33.62	2.36	36.16	3.82
