# Supplementary material for: Consequences of the Reproductive Effort of Dioecious Taxus baccata L. Females in a Generative Bud Removal Experiment—Important Role of Nitrogen in Female Reproduction
Source: Int J Mol Sci. 2022 Nov 17;23(22):14225. doi: 10.3390/ijms232214225 (PMC9695432; doi:10.3390/ijms232214225)
Supplement: Supplementary file 1 [file ijms-23-14225-s001.zip › Table S2.pdf]

Supplementary Table S2. ANOVA results for three comparisons of *Taxus baccata* shoots (divided into current and 1-year-old increments): control female vs. bud-removed female, bud-removed female vs. male and control female vs. male. Sex (or bud removal in comparison of female shoots), season and their interaction were used as sources of fixed effects. Individual and year of measurements nested in individuals were used as random effects. Results for parameters are shown in three sub-tables (A–C). F indicates female; M indicates male. P>0.05 is marked in bold.

A

	N current year			N one-year-old			C current year			C one-year-old			C: N current year			C: N one-year-old			soluble sugars current year			soluble sugars one-year-old			starch current year			starch one-year-old		
	DF	F Ratio	P	DF	F Ratio	P	DF	F Ratio	P	DF	F Ratio	P	DF	F Ratio	P	DF	F Ratio	P	DF	F Ratio	P	DF	F Ratio	P	DF	F Ratio	P	DF	F Ratio	P
**F control vs. F bud- removed**																														
season	3	53.69	**<.0001**	3	34.82	**<.0001**	3	3.34	**0.0229**	3	2.05	0.1135	3	51.50	**<.0001**	3	51.06	**<.0001**	3	7.78	**0.0001**	3	10.91	**<.0001**	3	237.63	**<.0001**	3	205.75	**<.0001**
bud removal	1	14.57	**0.0003**	1	1.30	0.2575	1	0.13	0.7235	1	1.27	0.2629	1	15.25	**0.0002**	1	5.36	**0.0233**	1	0.00	0.9892	1	0.73	0.3959	1	0.00	0.9462	1	2.05	0.1564
season x bud removal	3	0.60	0.6180	3	0.82	0.4878	3	1.65	0.1844	3	0.48	0.6974	3	0.05	0.9830	3	1.25	0.2958	3	0.42	0.7423	3	0.35	0.7905	3	0.38	0.7682	3	2.05	0.1137
**F bud- removed vs. M**																														
season	3	36.56	**<.0001**	3	34.82	**<.0001**	3	4.09	**0.0096**	3	2.21	0.0938	3	34.61	**<.0001**	3	42.62	**<.0001**	3	6.92	**0.0004**	3	11.03	**<.0001**	3	200.56	**<.0001**	3	183.22	**<.0001**
sex	1	2.28	0.1564	1	4.64	0.0540	1	0.25	0.6283	1	18.21	**0.0013**	1	1.25	0.2863	1	1.21	0.2939	1	0.05	0.8204	1	4.15	0.0698	1	3.06	0.1000	1	15.85	**0.0011**
season x sex	3	1.17	0.3273	3	0.31	0.8174	3	1.01	0.3938	3	2.58	0.0598	3	1.08	0.3622	3	0.25	0.8599	3	0.17	0.9179	3	1.04	0.3794	3	0.57	0.6356	3	0.17	0.9183
**F control vs. M**																														
season	3	27.05	**<.0001**	3	38.87	**<.0001**	3	0.85	0.4729	3	4.63	**0.0052**	3	31.02	**<.0001**	3	44.38	**<.0001**	3	14.92	**<.0001**	3	15.07	**<.0001**	3	209.71	**<.0001**	3	149.52	**<.0001**
sex	1	17.20	**0.0025**	1	7.58	**0.0274**	1	0.99	0.3428	1	5.03	**0.0474**	1	10.38	**0.0086**	1	6.67	**0.0318**	1	0.11	0.7458	1	0.58	0.4575	1	2.25	0.1523	1	3.01	0.1156
season x sex	3	0.95	0.4220	3	0.61	0.6130	3	0.57	0.6383	3	2.41	0.0740	3	0.89	0.4533	3	1.09	0.3617	3	0.81	0.4913	3	1.32	0.2743	3	0.27	0.8460	3	1.02	0.3894

B

	current year increment in two-year increments biomass (%)			specific leaf area current year			specific leaf area one-year-old			mean needle area current year			mean needle area one-year-old			mean needle length current year			mean needle length one-year-old			mean needle width current year			mean needle width one-year-old		
	DF	F Ratio	P	DF	F Ratio	P	DF	F Ratio	P	DF	F Ratio	P	DF	F Ratio	P	DF	F Ratio	P	DF	F Ratio	P	DF	F Ratio	P	DF	F Ratio	P
**F control vs. F bud-removed**																											
season	3	7.91	**<.0001**	3	44.30	**<.0001**	3	81.22	**<.0001**	3	5.15	**0.0021**	3	7.48	**0.0001**	3	4.48	**0.0050**	3	5.45	**0.0014**	3	3.54	**0.0165**	3	11.94	**<.0001**
bud removal	1	1.04	0.3099	1	0.00	0.9837	1	0.70	0.4045	1	0.04	0.8352	1	0.00	0.9806	1	0.35	0.5531	1	0.01	0.9226	1	0.81	0.3683	1	0.01	0.9066
season x bud removal	3	1.29	0.2805	3	1.35	0.2620	3	0.18	0.9113	3	1.03	0.3823	3	0.68	0.5647	3	1.00	0.3931	3	0.51	0.6781	3	0.86	0.4621	3	0.38	0.7647
**F bud- removed vs. M**																											
season	3	5.42	**0.0016**	3	55.27	**<.0001**	3	80.57	**<.0001**	3	0.49	0.6900	3	5.77	**0.0010**	3	0.45	0.7168	3	3.98	**0.0097**	3	0.17	0.9170	3	9.93	**<.0001**
sex	1	2.61	0.1233	1	10.33	**0.0047**	1	18.46	**0.0004**	1	1.50	0.2371	1	1.59	0.2233	1	1.30	0.2691	1	2.08	0.1663	1	0.48	0.4966	1	0.33	0.5739
season x sex	3	0.68	0.5640	3	1.03	0.3812	3	3.62	**0.0154**	3	2.21	0.0911	3	0.16	0.9214	3	2.51	0.0622	3	0.08	0.9729	3	1.07	0.3650	3	0.63	0.5976
**F control vs. M**																											
season	3	7.43	**0.0001**	3	35.20	**<.0001**	3	58.27	**<.0001**	3	2.69	**0.0499**	3	7.73	**<.0001**	3	2.32	0.0793	3	5.79	**0.0010**	3	1.99	0.1198	3	10.69	**<.0001**
sex	1	4.98	**0.0385**	1	9.52	**0.0063**	1	12.45	**0.0024**	1	1.67	0.2130	1	1.49	0.2380	1	1.73	0.2044	1	1.81	0.1954	1	0.21	0.6537	1	0.31	0.5872
season x sex	3	1.91	0.1321	3	0.27	0.8447	3	2.02	0.1155	3	3.76	**0.0128**	3	0.73	0.5386	3	2.89	**0.0387**	3	0.51	0.6754	3	3.02	**0.0327**	3	0.57	0.6385

C

	phenols current year			phenols one-year-old			tannins current year			tannins one-year-old		
	DF	F Ratio	P	DF	F Ratio	P	DF	F Ratio	P	DF	F Ratio	P
**F control vs. F bud-removed**												
season	3	3.30	**0.0240**	3	4.37	**0.0066**	3	7.56	**0.0002**	3	4.25	**0.0075**
bud removal	1	0.29	0.5896	1	0.38	0.5408	1	0.20	0.6597	1	0.20	0.6564
season x bud removal	3	1.64	0.1854	3	1.15	0.3342	3	0.70	0.5531	3	0.55	0.6474
**F bud- removed vs. M**												
season	3	2.84	**0.0438**	3	3.24	**0.0270**	3	18.64	**<.0001**	3	5.45	**0.0019**
sex	1	0.67	0.4238	1	6.49	**0.0247**	1	1.24	0.2798	1	0.00	0.9852
season x sex	3	0.97	0.4116	3	1.80	0.1545	3	3.19	**0.0287**	3	1.27	0.2917
**F control vs. M**												
season	3	0.72	0.5443	3	2.19	0.0959	3	11.69	**<.0001**	3	8.71	**<.0001**
sex	1	1.89	0.1882	1	6.89	**0.0215**	1	0.89	0.3588	1	0.03	0.8670
season x sex	3	1.59	0.1997	3	2.38	0.0764	3	2.57	0.0610	3	0.27	0.8465
